# Preserve or destroy: Orphan protein proteostasis and the heat shock response

**DOI:** 10.1083/jcb.202407123

**Published:** 2024-11-15

**Authors:** Asif Ali, Sarah Paracha, David Pincus

**Affiliations:** 1 Department of Molecular Genetics and Cell Biology, University of Chicago, Chicago, IL, USA; 2 Institute for Biophysical Dynamics, University of Chicago, Chicago, IL, USA; 3 Center for Physics of Evolution, University of Chicago, Chicago, IL, USA

## Abstract

Most eukaryotic genes encode polypeptides that are either obligate members of hetero-stoichiometric complexes or clients of organelle-targeting pathways. Proteins in these classes can be released from the ribosome as “orphans”—newly synthesized proteins not associated with their stoichiometric binding partner(s) and/or not targeted to their destination organelle. Here we integrate recent findings suggesting that although cells selectively degrade orphan proteins under homeostatic conditions, they can preserve them in chaperone-regulated biomolecular condensates during stress. These orphan protein condensates activate the heat shock response (HSR) and represent subcellular sites where the chaperones induced by the HSR execute their functions. Reversible condensation of orphan proteins may broadly safeguard labile precursors during stress.

## Teaser

Ali et al. offer a Perspective on how rather than degrading newly synthesized “orphan” proteins during stress, cells preserve them in condensates with chaperones that activate the heat shock response.

## Orphan protein quality control and the heat shock response (HSR)

Few proteins are islands. Most genes in the model eukaryote *Saccharomyces cerevisiae* encode proteins designated for specific trafficking to membrane-enclosed subcellular compartments or assembly with other cellular factors into stoichiometric complexes (Gavin et al., 2006; Krogan et al., 2006). Analyses of protein–protein interactions indicate that >50% of proteins have the propensity to engage in heteromeric complexes of defined stoichiometry, although membrane proteins tend to exhibit a slightly lower degree of interaction (Aebersold and Mann, 2016; Michaelis et al., 2023). Even under the most well-balanced homeostatic cellular conditions, the lack of operonic structure to eukaryotic genes and the inherent stochasticity of gene expression inevitably results in stoichiometric imbalances. Substituent polypeptides in a protein complex not associated with their binding partner(s), and newly synthesized membrane or organellar proteins not targeted to their destinations, are termed “orphan” proteins (Juszkiewicz and Hegde, 2018).

**Figure 1.**
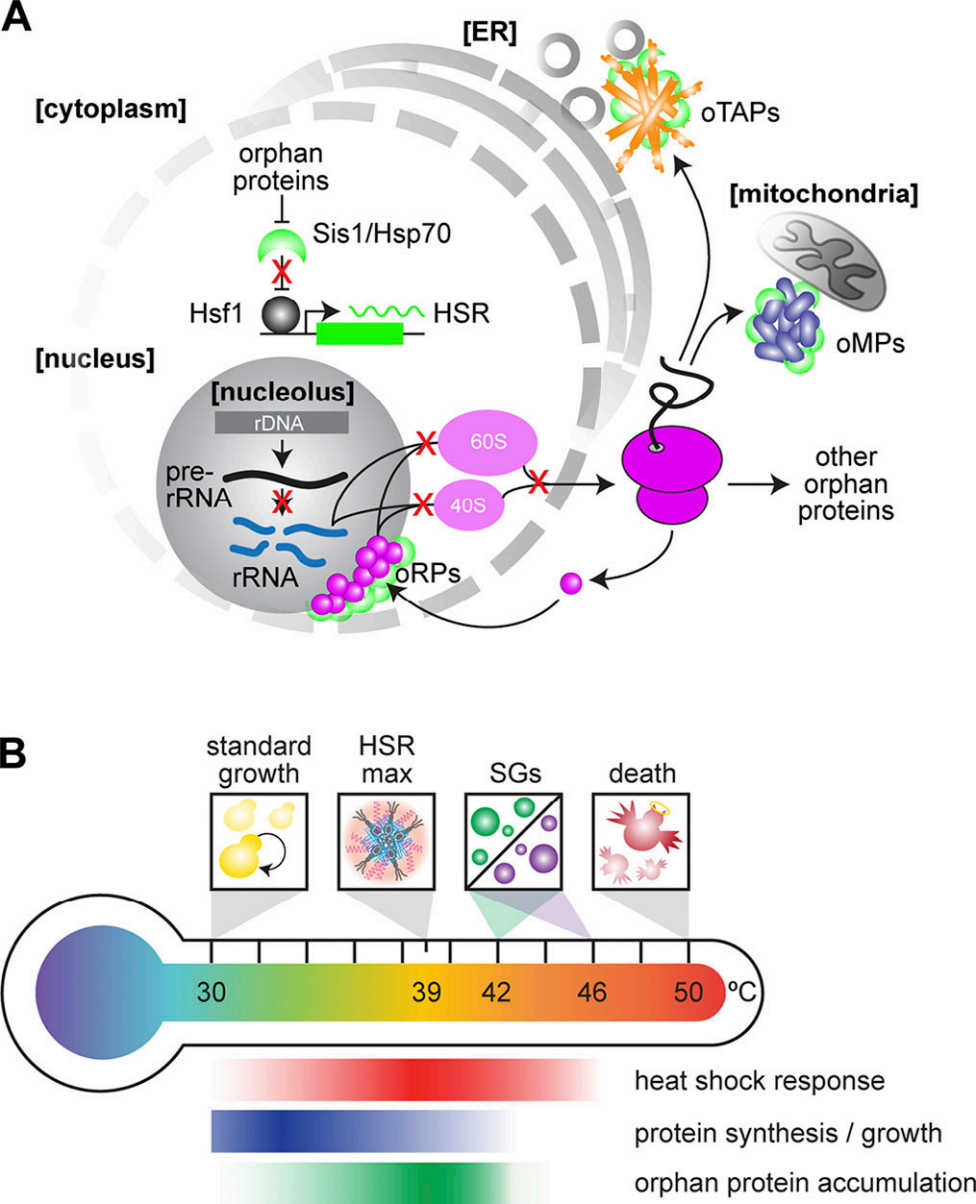
**Orphan proteins and the HSR. (A)** Accumulation of oRPs, oMPs, and oTAPs titrate the chaperone Hsp70 and its coregulator, the J-domain protein Sis1, away from Hsf1, activating the HSR. **(B)** Variation in the activity of the HSR, global protein synthesis, and the putative accumulation of orphan proteins as a function of temperature, highlighting key temperatures associated with growth, HSR maximal activity, condensation of different SG components, and cell death.

Under nonstress conditions, cells employ degradation mechanisms to recognize and remove orphan proteins, giving the cell a buffering capacity to counter small imbalances in stoichiometry. Even in aneuploid cells, in which protein complexes with members on different chromosomes have constitutive stoichiometric imbalances with substantial numbers of orphan proteins, protein homeostasis pathways maintain cell viability via degradation and adaptive aggregate/condensate formation (Ben-David and Amon, 2020; Brennan et al., 2019; Oromendia et al., 2012). However, saturation of this buffering capacity, through mutations or environmental perturbations, triggers an evolutionarily conserved transcriptional program known as the HSR (Alford et al., 2021; Brandman et al., 2012; Pincus, 2020) (Fig. 1 A). The HSR enhances the ability of cells to cope with the build-up of aggregation-prone orphan proteins by increasing the production of chaperones via the transcriptional activator Hsf1 (Dea and Pincus, 2024; Garde et al., 2024). Under prolonged stress, the HSR upregulates Rpn4, a transcription factor which in turn induces expression of the proteasomal machinery (Boos et al., 2019; Work and Brandman, 2021).

The observation that orphan proteins trigger cells to produce both chaperones and degradation machinery underscores the key regulatory decision that cells confront when orphans accumulate: to preserve or destroy them. Here, we will highlight recent discoveries describing dedicated proteostasis mechanisms that route orphans for degradation or preservation. In addition to specific ubiquitin–proteasome pathways to degrade different classes of orphan proteins under nonstress conditions, we discuss novel roles for the Hsp70 chaperone system in preserving orphan proteins in reversible biomolecular condensates during stress and how these orphan protein condensates serve as subcellular hubs that regulate—and are regulated by—the HSR (Fig. 1 A).

## Orphan protein degradation and accumulation during stress

Cells deploy specific ubiquitin–proteasome pathways to degrade different classes of orphan proteins. For example, cells use the mitochondrial protein translocation-associated degradation pathway and nuclear quality control factors to continuously survey the translocation through the outer membrane (TOM) complex and prevent clogging of the TOM channel with mitochondrial precursor proteins (Mårtensson et al., 2019; Shakya et al., 2021). When this safeguard is overwhelmed, such as via the acute blockage of mitochondrial import by engineered clogger proteins, orphan mitochondrial proteins (oMPs) accumulate in the cytosol in reversible condensates and selectively induce the HSR among all cellular stress responses (Boos et al., 2019; Krämer et al., 2023). Since these mitochondrial precursors require cytosolic chaperones to initiate targeting, their accumulation in the cytosol titrates Hsp70 away from repressing Hsf1 in the nucleus and thereby inducing the HSR (Feder et al., 2021; Krakowiak et al., 2018; Masser et al., 2019; Zheng et al., 2016) (Fig. 1 A). In further support of this mitochondria-to-HSR signaling axis, the mitochondrial unfolded protein response in mammalian cells was recently shown to be similarly triggered by oMPs accumulating in the cytosol and activating the HSR by Hsp70 sequestration (Sutandy et al., 2023).

The most heterotypic complex in the cell is the ribosome, which in eukaryotes requires the stoichiometric assembly of ∼80 different proteins and four RNAs (Lempiäinen and Shore, 2009; Shore and Albert, 2022; Shore et al., 2021). When there is a moderate excess of ribosomal protein production, such as when a single protein is overexpressed, the E3 ubiquitin ligase Tom1 targets the orphan ribosomal proteins (oRPs) for degradation by the proteasome (Sung et al., 2016a, 2016b). In human cells, HUWE1 and UBE2O have been implicated in degradation of orphan proteins including oRPs (Nguyen et al., 2017; Xu et al., 2016; Yanagitani et al., 2017). However, in cases when this clearance mechanism is inundated via genetic mutation or chemical perturbation, oRPs form nuclear condensates with chaperones and the ribosomal protein gene transcriptional activator Ifh1 (Albert et al., 2019; Tye et al., 2019). As when the oMP degradation system is overwhelmed, accumulated oRPs activate the HSR by sequestering Hsp70 (Albert et al., 2019; Ali et al., 2023; Tye et al., 2019) (Fig. 1 A).

Tail-anchored proteins represent a third class of orphan proteins known to activate the HSR. Like mitochondrial import, tail anchor membrane insertion is mediated by a chaperone cascade that begins with Hsp70 delivering clients to the guided entry of tail-anchored proteins (GET) pathway (Brandman et al., 2012; Cho et al., 2024; Shan, 2019; Wang et al., 2014). Glucose depletion and deletion of GET pathway factors result in accumulation of orphan tail-anchored proteins (oTAPs) in cytosolic clusters that in current parlance would be termed condensates (Powis et al., 2013; Wang et al., 2014). While it remains unclear whether these oTAP condensates form as HSR signaling hubs during physiological heat shock, their formation upon glucose depletion—a condition known to activate the HSR (Hahn and Thiele, 2004; Zid and O’Shea, 2014)—suggests that they may serve as physiological HSR activators under some conditions (Fig. 1 A).

In addition to oMPs, oRPs, and oTAPs, other classes of orphan proteins have been found to have dedicated E3 ligases to enforce stoichiometries in mammalian cells, including kinases, transcription factors, the chaperonin containing t-complex protein (CCT) complex, and the proteasome itself (Mark et al., 2023; Mena et al., 2018; Padovani et al., 2022; Yagita et al., 2023; Zavodszky et al., 2021). Many of these proteins fold on their own, so their accumulation may not activate the HSR. By contrast, defective ribosome products, a terminal class of orphan proteins, are misfolded by definition and can translocate to the nucleus and accumulate at the nucleolus (Davis et al., 2021; Mediani et al., 2019). Whether these additional classes of orphan proteins form condensates and activate the HSR during stress is not currently known.

## Orphan protein condensates as physiological activators of the HSR

The stress conditions under which orphan protein condensates may form to signal the HSR, while potentially numerous, are physically constrained. Heat shock, as perhaps the best studied environmental stress, presents an illustrative example. Yeast cells grow readily at 30°C with no signatures of stress evident in the transcriptome or proteome, and they die by lysis after a few minutes at 50°C. As the temperature rises above the standard growth condition, i.e., as the magnitude of stress increases, the HSR is induced, while overall protein synthesis and growth are concomitantly repressed. The magnitude of the HSR peaks at 39°C, when Hsf1 forms transcriptional condensates, and begins to decrease as the temperature is further increased due to the formation of stress granules (SGs) that enforce the shutdown of translation (Iserman et al., 2020; Chowdhary et al., 2022; Kik et al., 2024; Riback et al., 2017) (Fig. 1 B). This loss of HSR output at temperatures above which SGs form and global translation is reduced is consistent with observations that Hsf1 activity is also impaired at mild and moderate heat shock if translation is inhibited by cycloheximide or rocaglamide treatment or amino acid depletion (Masser et al., 2019; Santagata et al., 2013; Triandafillou et al., 2020; Tye and Churchman, 2021). Since production of orphan proteins requires translation, this tight correlation of HSR output and ongoing protein synthesis further supports the notion that orphan protein accumulation activates the HSR during physiological stress.

## Reversible condensation of labile proteins in the nucleus during stress

While initially generated by cytosolic ribosomes, oRPs—which constitute a substantial fraction of all orphan proteins due to their high abundance—are subsequently imported into the nucleus for assembly with rRNA in the nucleolus. With the help a nuclear-specific proteostasis network and specialized phase-separated sites devoted to quality control, the nucleus helps maintain the many metastable proteins that make up its proteome (Miller et al., 2015a, 2015b; Park et al., 2013; Prasad et al., 2018; Samant et al., 2018; Sontag et al., 2017, 2023).

The most prominent membrane-free compartment in the nucleus is the nucleolus, the site of ribosome biogenesis (Feric et al., 2016; Lafontaine et al., 2021). The outer granular component (GC) of the nucleolus in human cells is densely packed with the negatively charged protein nucleophosmin (NPM1) that scaffolds the phase separation of the GC and has long been implicated in cancer (Grisendi et al., 2006; Mitrea et al., 2018). This GC has been found to host ∼200 stress-sensitive proteins during acute heat shock, affording these proteins from both outside and within the nucleolus protection during stress (Frottin et al., 2019). Hsp70 and cofactors localize to this site as well, maintaining proteins in a soluble state until stress recovery, suggesting an adaptive role for the nucleolar compartment. If the cells undergo prolonged stress, the GC solidifies and is no longer able to carry out its proteostasis function (Frottin et al., 2019).

Similarly, in *S. pombe* cells during heat stress, nuclear and nucleolar proteins segregate to the nucleolar periphery and rearrange to form rings. The rings serve to isolate essential proteins required for cellular transcription, processing, and cell cycle regulators, thereby inhibiting and protecting these proteins during acute stress (Gallardo et al., 2020). Upon release from heat stress, the nucleolar rings disassembled, allowing the proteins to revert to their previous locations. Hsp70 is present in these nucleolar rings during heat shock, and the disaggregase Hsp104 was found to be required for the efficient dissolution of these rings upon recovery (Cabrera et al., 2020; Gallardo et al., 2020).

During nutrient depletion, budding yeast cells enter a quiescent state where cells exit the cell cycle, translation rates decline and there is oxidative metabolic activity, essentially placing the cells in a state of stress as they must work to protect their proteomes and maintain function (Sagot and Laporte, 2019; Sun and Gresham, 2021). Cellular reprogramming following nutrient depletion involves the assembly of reversible cytosolic biomolecular condensates including SGs and processing (P)-bodies, long thought to be sites of mRNA storage and degradation (Decker and Parker, 2012). Multiple studies have identified the presence of SGs and P-bodies in the cytosol that sequester and maintain essential components of the proteostasis and translation machinery during quiescence, allowing them to be reactivated when protein synthesis is once again resumed (Coller, 2011; Grousl et al., 2022; Liu et al., 2012; Marshall and Vierstra, 2018; Protter and Parker, 2016). Recently, the nucleus has also been shown to harbor reversible clusters of translation-associated proteins during prolonged stress and starvation (Kohler et al., 2024). Hsp104 likewise accumulates in the nucleus, safeguarding these factors for the rapid restart of protein synthesis upon refeeding. These nuclear localized translation factors qualify as orphan proteins due to their localization away from their functional home in the cytosol, and the chaperone-associated condensates they form preserve them rather than facilitating degradation.

## Preservation of oRPs in stress-induced condensates

Cells regulate the rate of ribosome production according to nutritional cues, and ribosomal protein gene transcription by RNA Pol II is tightly coordinated with rRNA synthesis by RNA Pol I (Lempiäinen and Shore, 2009; Shore and Albert, 2022; Shore et al., 2021; Woolford and Baserga, 2013). Across a wide range of environmental conditions, a common transcriptional response to the stress is to repress transcription of ribosomal RNA (rRNA) and mRNAs encoding ribosomal proteins and biogenesis factors (Gasch et al., 2000; Gasch and Werner-Washburne, 2002). However, as described above in the case of case of heat shock, protein translation remains active at intermediate levels of stress (Iserman et al., 2020; Mühlhofer et al., 2019). To the extent that preexisting mRNAs encoding ribosomal proteins continue to be translated during stress, they produce oRPs as there is no rRNA for them to bind to. In the absence of rRNA, oRPs require chaperones and nuclear import factors to maintain their solubility and prevent aggregation (Pillet et al., 2022; Seidel et al., 2023; Tye et al., 2019).

**Figure 2.**
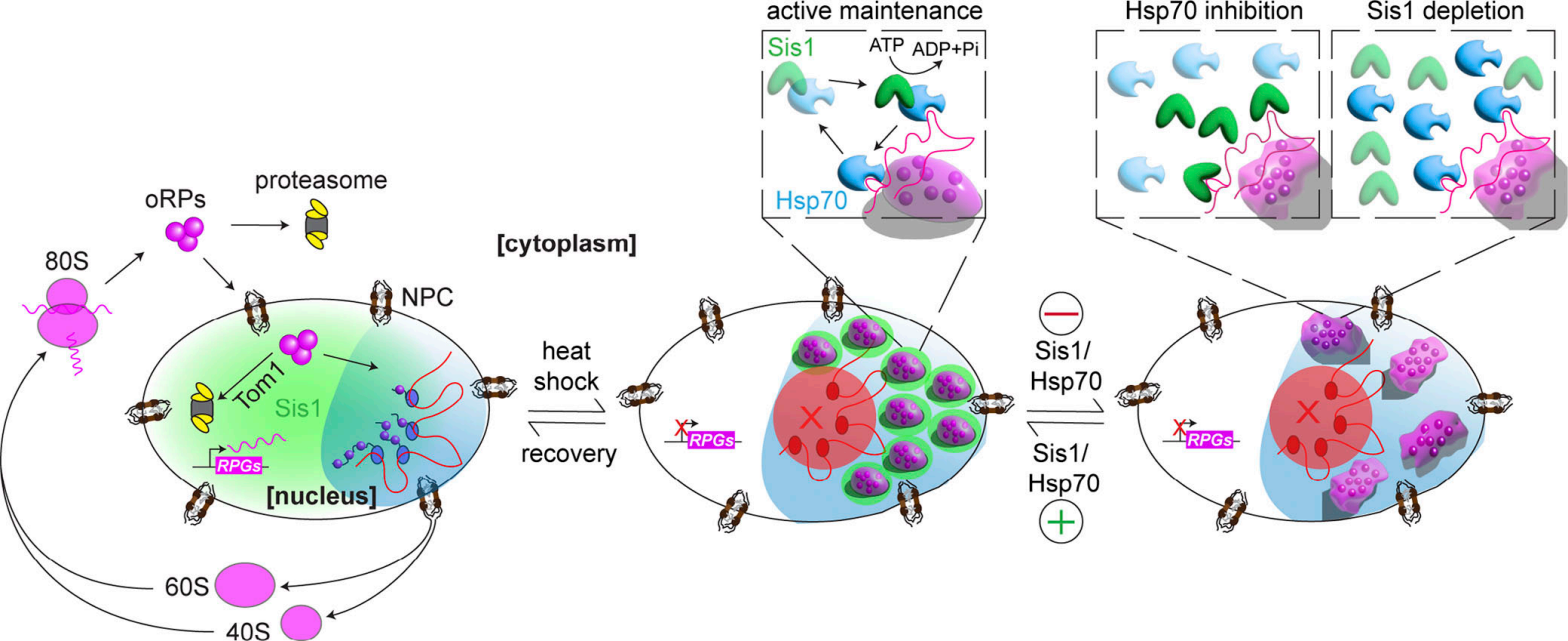
**Preservation of oRPs in chaperone-regulated condensates.** The ribosome biogenesis cycle is disrupted during stress, resulting in the formation of oRP condensates at the nucleolar periphery, the reversibility of which is maintained by the activity of Hsp70 and Sis1. Even solid oRP condensates are reversible if Sis1 and Hsp70 are allowed to resume their activity. NPC, nuclear pore complex; RPG, ribosomal protein gene.

As described above, cells target oRPs for proteasomal degradation when expressed ectopically (Sung et al., 2016a, 2016b). By contrast, during heat shock, endogenous oRPs form adaptive, reversible condensates localized to the outer region of the nucleolus in yeast and human cells that preserve the oRPs for usage once the cell is no longer under stress (Ali et al., 2023) (Fig. 2). Although it is unclear why oRPs have distinct fates in these conditions, it could be in part due to the number of molecules: a single overexpressed protein can be degraded, but a sudden accumulation of all ∼80 oRPs may overwhelm the ubiquitin proteasome system.

As with previously described adaptive condensates such as SGs, oRP condensates interact with chaperone proteins, most prominently the J-domain protein (JDP) Sis1/DnaJB6 and Hsp70. Sis1 is an essential JDP and co-chaperone for Hsp70 required for biogenesis of mTORC1-like kinase complexes and for partial repression of the HSR under nonstress conditions by targeting Hsp70 to bind to Hsf1 (Feder et al., 2021; Garde et al., 2023; Klaips et al., 2020; Luke et al., 1991; Schilke and Craig, 2022). When Sis1 was depleted or Hsp70 was inhibited, oRP condensates solidified, showing that these chaperones are required for the maintenance of the dynamic state of these condensates. Recently, the ubiquitin-related modifier protein Urm1 was shown to localize to peri-nucleolar and cytosolic condensates during stress, promote the reversibility of SGs, and preserve labile proteins (Cairo et al., 2024). Thus, the Sis1/Hsp70 chaperone system may collaborate with other proteostasis mechanisms to modulate condensate dynamics. Without Sis1 or Hsp70, the reversibility of oRP condensates was delayed, and recovery from heat shock and resumption of cell growth was postponed (Fig. 2). This example represents a case where the biophysical properties of the oRP condensates—the liquid-like fluidity enforced by interactions with Sis1 and Hsp70—serve an adaptive advantage of preserving oRP functionality, allowing them to be readily incorporated into nascent ribosomes once rRNA synthesis resumes (Ali et al., 2023).

**Figure 3.**
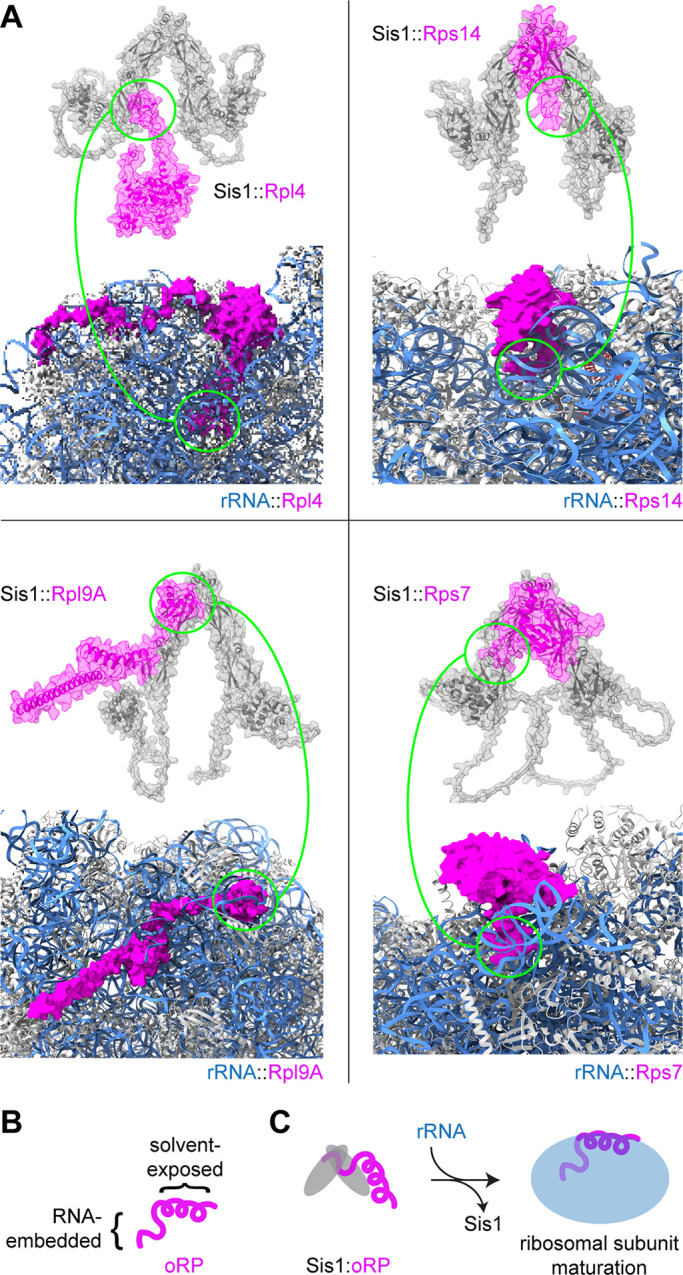
**Predicted structures of Sis1 interacting with oRPs. (A)** AlphaFold Multimer structure of Sis1 dimers with oRPs depicted above the region of the ribosome containing each mature ribosomal protein (Evans et al., 2021, *Preprint*). These RPs are each integrated into the ribosome at different assembly steps. Sis1 dimers shown in gray; oRPs shown in magenta; rRNA shown in blue. The regions predicted to interact with Sis1, circled in green, are buried in rRNA in the mature ribosome. Importantly, these structures represent only single poses predicted by the model. These are almost certainly not the only way in which Sis1 can interact with these proteins. **(B)** Schematic of a single oRP. **(C)** Schematic for how RPs bind in a mutually exclusive manner to Sis1 and rRNA.

Using AlphaFold Multimer (Evans et al., 2021, *Preprint*), we have generated model structures of the complex of Sis1 dimers with four different oRPs to guide our molecular interpretation (Fig. 3 A). Importantly, these artificial intelligence–based models do not necessarily represent reality and have not been experimentally validated. With these models, we do not intend to claim that any specific residues form binding interfaces with Sis1—it is likely that Sis1 interacts with these proteins in multiple configurations. Rather, we generated these models to determine whether Sis1 may recognize any common features on the different oRPs. Two of the modeled oRPs are constituents of the 60S subunit, two of the 40S, and each of the four are incorporated at different ribosome assembly steps (Woolford and Baserga, 2013). The models uniformly predicted that Sis1 interacts with regions of the oRPs that would be buried away from the surface of the ribosome and the surrounding solvent by interacting directly with rRNA (Fig. 3 B). These regions where Sis1 is predicted to bind are also predicted to interact with Hsp70 (Rüdiger et al., 2001). This suggests a simple mechanism by which the reappearance of rRNA would outcompete Sis1 and Hsp70 to release the oRPs from the chaperones to resume ribosome biogenesis (Fig. 3 C). Notably, this molecular logic of oRP recognition by Sis1—binding to the surfaces buried in the mature complex—is analogous to how the ubiquitin ligases recognize orphan proteins for degradation (Juszkiewicz and Hegde, 2018; Padovani et al., 2022).

## Outlook: Who needs a chaperone more than an orphan?

In this perspective, we draw a through line connecting orphan proteins, stress-induced adaptive condensates, and the HSR. Since the discovery of the HSR, the endogenous signals that activate the response following heat shock and other stressors were long presumed to be toxic aggregates of denatured proteins (Lindquist, 1986; Zheng et al., 2016). However, much recent work has provided evidence in support of a different paradigm in which the protein aggregates that form during heat shock are nontoxic and programmed by evolution as adaptive mechanisms under specific conditions (Franzmann et al., 2018; Iserman et al., 2020; Riback et al., 2017; Wallace et al., 2015; Yoo et al., 2019).

From the perspective of HSR activation, two major classes of proteins have been implicated as the physiological ligands: heat- and pH-dependent condensates such as SGs and newly synthesized proteins (Garde et al., 2023; Santiago et al., 2020; Triandafillou et al., 2020; Tye and Churchman, 2021). As oRPs have directly been shown to be a subset of the newly synthesized proteins that activate the HSR (Ali et al., 2023), and oMPs and oTAPs have been strongly implicated (Boos et al., 2019; Brandman et al., 2012; Krämer et al., 2023; Wang et al., 2014), we speculate that different classes of orphan proteins will serve as physiological ligands of the HSR across diverse conditions. From the perspective of the orphan proteins, the chaperones provide binding partners and protection against promiscuous interactions. Moreover, the condensates they form function as nurseries to provide distributed coverage for precursor proteins by a limited number of chaperone proteins, signaling the HSR to increase chaperone production all the while. The notion that newly synthesized proteins are being “chaperoned” in an “orphanage” to conserve limited resources during stress is a powerful metaphor to describe these recently discovered preservation mechanisms.

## References

[bib1] Aebersold, R., and M. Mann. 2016. Mass-spectrometric exploration of proteome structure and function. Nature. 537:347–355. 10.1038/nature19949 27629641

[bib2] Albert, B., I.C. Kos-Braun, A.K. Henras, C. Dez, M.P. Rueda, X. Zhang, O. Gadal, M. Kos, and D. Shore. 2019. A ribosome assembly stress response regulates transcription to maintain proteome homeostasis. Elife. 8:e45002. 10.7554/eLife.45002 31124783 PMC6579557

[bib3] Alford, B.D., E. Tassoni-Tsuchida, D. Khan, J.J. Work, G. Valiant, and O. Brandman. 2021. ReporterSeq reveals genome-wide dynamic modulators of the heat shock response across diverse stressors. Elife. 10:e57376. 10.7554/eLife.57376 34223816 PMC8257254

[bib4] Ali, A., R. Garde, O.C. Schaffer, J.A.M. Bard, K. Husain, S.K. Kik, K.A. Davis, S. Luengo-Woods, M.G. Igarashi, D.A. Drummond, . 2023. Adaptive preservation of orphan ribosomal proteins in chaperone-dispersed condensates. Nat. Cell Biol. 25:1691–1703. 10.1038/s41556-023-01253-2 37845327 PMC10868727

[bib5] Ben-David, U., and A. Amon. 2020. Context is everything: Aneuploidy in cancer. Nat. Rev. Genet. 21:44–62. 10.1038/s41576-019-0171-x 31548659

[bib6] Boos, F., L. Krämer, C. Groh, F. Jung, P. Haberkant, F. Stein, F. Wollweber, A. Gackstatter, E. Zöller, M. van der Laan, . 2019. Mitochondrial protein-induced stress triggers a global adaptive transcriptional programme. Nat. Cell Biol. 21:442–451. 10.1038/s41556-019-0294-5 30886345

[bib7] Brandman, O., J. Stewart-Ornstein, D. Wong, A. Larson, C.C. Williams, G.W. Li, S. Zhou, D. King, P.S. Shen, J. Weibezahn, . 2012. A ribosome-bound quality control complex triggers degradation of nascent peptides and signals translation stress. Cell. 151:1042–1054. 10.1016/j.cell.2012.10.044 23178123 PMC3534965

[bib8] Brennan, C.M., L.P. Vaites, J.N. Wells, S. Santaguida, J.A. Paulo, Z. Storchova, J.W. Harper, J.A. Marsh, and A. Amon. 2019. Protein aggregation mediates stoichiometry of protein complexes in aneuploid cells. Genes Dev. 33:1031–1047. 10.1101/gad.327494.119 31196865 PMC6672052

[bib9] Cabrera, M., S. Boronat, L. Marte, M. Vega, P. Perez, J. Ayte, and E. Hidalgo. 2020. Chaperone-facilitated aggregation of thermo-sensitive proteins shields them from degradation during heat stress. Cell Rep. 30:2430–2443.e4. 10.1016/j.celrep.2020.01.077 32075773

[bib10] Cairo, L.V., X. Hong, M.B. Müller, P. Yuste-Checa, C. Jagadeesan, A. Bracher, S.-H. Park, M. Hayer-Hartl, and F.U. Hartl. 2024. Stress-dependent condensate formation regulated by the ubiquitin-related modifier Urm1. Cell. 187:4656–4673.e28. 10.1016/j.cell.2024.06.009 38942013

[bib11] Cho, H., Y. Liu, S. Chung, S. Chandrasekar, S. Weiss, and S.O. Shan. 2024. Dynamic stability of Sgt2 enables selective and privileged client handover in a chaperone triad. Nat. Commun. 15:134. 10.1038/s41467-023-44260-5 38167697 PMC10761869

[bib12] Chowdhary, S., A.S. Kainth, S. Paracha, D.S. Gross, and D. Pincus. 2022. Inducible transcriptional condensates drive 3D genome reorganization in the heat shock response. Mol. Cell. 82:4386–4399.e7. 10.1016/j.molcel.2022.10.013 36327976 PMC9701134

[bib13] Coller, H.A. 2011. Cell biology. The essence of quiescence. Science. 334:1074–1075. 10.1126/science.1216242 22116874 PMC4765170

[bib14] Davis, Z.H., L. Mediani, F. Antoniani, J. Vinet, S. Li, S. Alberti, B. Lu, A.S. Holehouse, S. Carra, and O. Brandman. 2021. Protein products of nonstop mRNA disrupt nucleolar homeostasis. Cell Stress Chaperones. 26:549–561. 10.1007/s12192-021-01200-w 33619693 PMC8065075

[bib95] Dea, A., and D. Pincus. 2024. The heat shock response as a condensate cascade. J. Mol. Biol. 436. 168642. 10.1016/j.jmb.2024.168642 38848866 PMC11214683

[bib15] Decker, C.J., and R. Parker. 2012. P-bodies and stress granules: Possible roles in the control of translation and mRNA degradation. Cold Spring Harb. Perspect. Biol. 4:a012286. 10.1101/cshperspect.a012286 22763747 PMC3428773

[bib16] Evans, R., M. O’Neill, A. Pritzel, N. Antropova, A. Senior, T. Green, A. Žídek, R. Bates, S. Blackwell, and J. Yim. 2021. Protein complex prediction with AlphaFold-Multimer. bioRxiv. 10.1101/2021.10.04.463034 (Preprint posted April 10, 2021).

[bib17] Feder, Z.A., A. Ali, A. Singh, J. Krakowiak, X. Zheng, V.P. Bindokas, D. Wolfgeher, S.J. Kron, and D. Pincus. 2021. Subcellular localization of the J-protein Sis1 regulates the heat shock response. J. Cell Biol. 220:e202005165. 10.1083/jcb.202005165 PMC7748816 33326013

[bib18] Feric, M., N. Vaidya, T.S. Harmon, D.M. Mitrea, L. Zhu, T.M. Richardson, R.W. Kriwacki, R.V. Pappu, and C.P. Brangwynne. 2016. Coexisting liquid phases underlie nucleolar subcompartments. Cell. 165:1686–1697. 10.1016/j.cell.2016.04.047 27212236 PMC5127388

[bib19] Franzmann, T.M., M. Jahnel, A. Pozniakovsky, J. Mahamid, A.S. Holehouse, E. Nüske, D. Richter, W. Baumeister, S.W. Grill, R.V. Pappu, . 2018. Phase separation of a yeast prion protein promotes cellular fitness. Science. 359:eaao5654. 10.1126/science.aao5654 29301985

[bib20] Frottin, F., F. Schueder, S. Tiwary, R. Gupta, R. Körner, T. Schlichthaerle, J. Cox, R. Jungmann, F.U. Hartl, and M.S. Hipp. 2019. The nucleolus functions as a phase-separated protein quality control compartment. Science. 365:342–347. 10.1126/science.aaw9157 31296649

[bib21] Gallardo, P., P. Real-Calderón, I. Flor-Parra, S. Salas-Pino, and R.R. Daga. 2020. Acute heat stress leads to reversible aggregation of nuclear proteins into nucleolar rings in fission yeast. Cell Rep. 33:108377. 10.1016/j.celrep.2020.108377 33176152

[bib94] Garde, R., A. Dea, M.F. Herwig, A. Ali, and D. Pincus. 2024. Feedback control of the heat shock response by spatiotemporal regulation of Hsp70. J. Cell Biol. 223. e202401082. 10.1083/jcb.202401082 39302312 PMC11415305

[bib22] Garde, R., A. Singh, A. Ali, and D. Pincus. 2023. Transcriptional regulation of Sis1 promotes fitness but not feedback in the heat shock response. Elife. 12:e79444. 10.7554/eLife.79444 37158601 PMC10191621

[bib23] Gasch, A.P., P.T. Spellman, C.M. Kao, O. Carmel-Harel, M.B. Eisen, G. Storz, D. Botstein, and P.O. Brown. 2000. Genomic expression programs in the response of yeast cells to environmental changes. Mol. Biol. Cell. 11:4241–4257. 10.1091/mbc.11.12.4241 11102521 PMC15070

[bib24] Gasch, A.P., and M. Werner-Washburne. 2002. The genomics of yeast responses to environmental stress and starvation. Funct. Integr. Genomics. 2:181–192. 10.1007/s10142-002-0058-2 12192591

[bib25] Gavin, A.-C., P. Aloy, P. Grandi, R. Krause, M. Boesche, M. Marzioch, C. Rau, L.J. Jensen, S. Bastuck, B. Dümpelfeld, . 2006. Proteome survey reveals modularity of the yeast cell machinery. Nature. 440:631–636. 10.1038/nature04532 16429126

[bib26] Grisendi, S., C. Mecucci, B. Falini, and P.P. Pandolfi. 2006. Nucleophosmin and cancer. Nat. Rev. Cancer. 6:493–505. 10.1038/nrc1885 16794633

[bib27] Grousl, T., J. Vojtova, J. Hasek, and T. Vomastek. 2022. Yeast stress granules at a glance. Yeast. 39:247–261. 10.1002/yea.3681 34791685

[bib28] Hahn, J.-S., and D.J. Thiele. 2004. Activation of the Saccharomyces cerevisiae heat shock transcription factor under glucose starvation conditions by Snf1 protein kinase. J. Biol. Chem. 279:5169–5176. 10.1074/jbc.M311005200 14612437

[bib29] Iserman, C., C. Desroches Altamirano, C. Jegers, U. Friedrich, T. Zarin, A.W. Fritsch, M. Mittasch, A. Domingues, L. Hersemann, M. Jahnel, . 2020. Condensation of Ded1p promotes a translational switch from housekeeping to stress protein production. Cell. 181:818–831.e19. 10.1016/j.cell.2020.04.009 32359423 PMC7237889

[bib30] Juszkiewicz, S., and R.S. Hegde. 2018. Quality control of orphaned proteins. Mol. Cell. 71:443–457. 10.1016/j.molcel.2018.07.001 30075143 PMC6624128

[bib96] Kik, S.K., D. Christopher, H. Glauninger, C.W. Hickernell, J.A.M. Bard, K.M. Lin, A.H. Squires, M. Ford, T.R. Sosnick, and D.A. Drummond. 2024. An adaptive biomolecular condensation response is conserved across environmentally divergent species. Nat. Commun. 15. 3127. 10.1038/s41467-024-47355-9 38605014 PMC11009240

[bib32] Klaips, C.L., M.H.M. Gropp, M.S. Hipp, and F.U. Hartl. 2020. Sis1 potentiates the stress response to protein aggregation and elevated temperature. Nat. Commun. 11:6271. 10.1038/s41467-020-20000-x 33293525 PMC7722728

[bib33] Kohler, V., A. Kohler, L.L. Berglund, X. Hao, S. Gersing, A. Imhof, T. Nyström, J.L. Höög, M. Ott, C. Andréasson, and S. Büttner. 2024. Nuclear Hsp104 safeguards the dormant translation machinery during quiescence. Nat. Commun. 15:315. 10.1038/s41467-023-44538-8 38182580 PMC10770042

[bib34] Krakowiak, J., X. Zheng, N. Patel, Z.A. Feder, J. Anandhakumar, K. Valerius, D.S. Gross, A.S. Khalil, and D. Pincus. 2018. Hsf1 and Hsp70 constitute a two-component feedback loop that regulates the yeast heat shock response. Elife. 7:e31668. 10.7554/eLife.31668 29393852 PMC5809143

[bib35] Krämer, L., N. Dalheimer, M. Räschle, Z. Storchová, J. Pielage, F. Boos, and J.M. Herrmann. 2023. MitoStores: Chaperone-controlled protein granules store mitochondrial precursors in the cytosol. EMBO J. 42:e112309. 10.15252/embj.2022112309 36704946 PMC10068336

[bib36] Krogan, N.J., G. Cagney, H. Yu, G. Zhong, X. Guo, A. Ignatchenko, J. Li, S. Pu, N. Datta, A.P. Tikuisis, . 2006. Global landscape of protein complexes in the yeast Saccharomyces cerevisiae. Nature. 440:637–643. 10.1038/nature04670 16554755

[bib37] Lafontaine, D.L.J., J.A. Riback, R. Bascetin, and C.P. Brangwynne. 2021. The nucleolus as a multiphase liquid condensate. Nat. Rev. Mol. Cell Biol. 22:165–182. 10.1038/s41580-020-0272-6 32873929

[bib38] Lempiäinen, H., and D. Shore. 2009. Growth control and ribosome biogenesis. Curr. Opin. Cell Biol. 21:855–863. 10.1016/j.ceb.2009.09.002 19796927

[bib39] Lindquist, S. 1986. The heat-shock response. Annu. Rev. Biochem. 55:1151–1191. 10.1146/annurev.bi.55.070186.005443 2427013

[bib40] Liu, I.-C., S.-W. Chiu, H.-Y. Lee, and J.-Y. Leu. 2012. The histone deacetylase Hos2 forms an Hsp42-dependent cytoplasmic granule in quiescent yeast cells. Mol. Biol. Cell. 23:1231–1242. 10.1091/mbc.e11-09-0752 22337769 PMC3315813

[bib41] Luke, M.M., A. Sutton, and K.T. Arndt. 1991. Characterization of SIS1, a Saccharomyces cerevisiae homologue of bacterial dnaJ proteins. J. Cell Biol. 114:623–638. 10.1083/jcb.114.4.623 1714460 PMC2289895

[bib42] Mark, K.G., S. Kolla, J.D. Aguirre, D.M. Garshott, S. Schmitt, D.L. Haakonsen, C. Xu, L. Kater, G. Kempf, and B. Martínez-González. 2023. Orphan quality control shapes network dynamics and gene expression. Cell. 186:3460–3475.e23. 10.1016/j.cell.2023.06.015 37478862

[bib43] Marshall, R.S., and R.D. Vierstra. 2018. Proteasome storage granules protect proteasomes from autophagic degradation upon carbon starvation. Elife. 7:e34532. 10.7554/eLife.34532 29624167 PMC5947986

[bib44] Mårtensson, C.U., C. Priesnitz, J. Song, L. Ellenrieder, K.N. Doan, F. Boos, A. Floerchinger, N. Zufall, S. Oeljeklaus, B. Warscheid, and T. Becker. 2019. Mitochondrial protein translocation-associated degradation. Nature. 569:679–683. 10.1038/s41586-019-1227-y 31118508

[bib45] Masser, A.E., W. Kang, J. Roy, J. Mohanakrishnan Kaimal, J. Quintana-Cordero, M.R. Friedländer, and C. Andréasson. 2019. Cytoplasmic protein misfolding titrates Hsp70 to activate nuclear Hsf1. Elife. 8:e47791. 10.7554/eLife.47791 31552827 PMC6779467

[bib46] Mediani, L., J. Guillén-Boixet, J. Vinet, T.M. Franzmann, I. Bigi, D. Mateju, A.D. Carrà, F.F. Morelli, T. Tiago, I. Poser, . 2019. Defective ribosomal products challenge nuclear function by impairing nuclear condensate dynamics and immobilizing ubiquitin. EMBO J. 38:e101341. 10.15252/embj.2018101341 31271238 PMC6669919

[bib47] Mena, E.L., R.A.S. Kjolby, R.A. Saxton, A. Werner, B.G. Lew, J.M. Boyle, R. Harland, and M. Rape. 2018. Dimerization quality control ensures neuronal development and survival. Science. 362:eaap8236. 10.1126/science.aap8236 30190310

[bib48] Michaelis, A.C., A.-D. Brunner, M. Zwiebel, F. Meier, M.T. Strauss, I. Bludau, and M. Mann. 2023. The social and structural architecture of the yeast protein interactome. Nature. 624:192–200. 10.1038/s41586-023-06739-5 37968396 PMC10700138

[bib49] Miller, S.B., C.T. Ho, J. Winkler, M. Khokhrina, A. Neuner, M.Y. Mohamed, D.L. Guilbride, K. Richter, M. Lisby, E. Schiebel, . 2015a. Compartment-specific aggregases direct distinct nuclear and cytoplasmic aggregate deposition. EMBO J. 34:778–797. 10.15252/embj.201489524 25672362 PMC4369314

[bib50] Miller, S.B., A. Mogk, and B. Bukau. 2015b. Spatially organized aggregation of misfolded proteins as cellular stress defense strategy. J. Mol. Biol. 427:1564–1574. 10.1016/j.jmb.2015.02.006 25681695

[bib51] Mitrea, D.M., J.A. Cika, C.B. Stanley, A. Nourse, P.L. Onuchic, P.R. Banerjee, A.H. Phillips, C.-G. Park, A.A. Deniz, and R.W. Kriwacki. 2018. Self-interaction of NPM1 modulates multiple mechanisms of liquid-liquid phase separation. Nat. Commun. 9:842. 10.1038/s41467-018-03255-3 29483575 PMC5827731

[bib52] Mühlhofer, M., E. Berchtold, C.G. Stratil, G. Csaba, E. Kunold, N.C. Bach, S.A. Sieber, M. Haslbeck, R. Zimmer, and J. Buchner. 2019. The heat shock response in yeast maintains protein homeostasis by chaperoning and replenishing proteins. Cell Rep. 29:4593–4607.e8. 10.1016/j.celrep.2019.11.109 31875563

[bib53] Nguyen, A.T., M.A. Prado, P.J. Schmidt, A.K. Sendamarai, J.T. Wilson-Grady, M. Min, D.R. Campagna, G. Tian, Y. Shi, V. Dederer, . 2017. UBE2O remodels the proteome during terminal erythroid differentiation. Science. 357:eaan0218. 10.1126/science.aan0218 28774900 PMC5812729

[bib54] Oromendia, A.B., S.E. Dodgson, and A. Amon. 2012. Aneuploidy causes proteotoxic stress in yeast. Genes Dev. 26:2696–2708. 10.1101/gad.207407.112 23222101 PMC3533075

[bib55] Padovani, C., P. Jevtić, and M. Rapé. 2022. Quality control of protein complex composition. Mol. Cell. 82:1439–1450. 10.1016/j.molcel.2022.02.029 35316660

[bib56] Park, S.H., Y. Kukushkin, R. Gupta, T. Chen, A. Konagai, M.S. Hipp, M. Hayer-Hartl, and F.U. Hartl. 2013. PolyQ proteins interfere with nuclear degradation of cytosolic proteins by sequestering the Sis1p chaperone. Cell. 154:134–145. 10.1016/j.cell.2013.06.003 23791384

[bib57] Pillet, B., A. Méndez-Godoy, G. Murat, S. Favre, M. Stumpe, L. Falquet, and D. Kressler. 2022. Dedicated chaperones coordinate co-translational regulation of ribosomal protein production with ribosome assembly to preserve proteostasis. Elife. 11:e74255. 10.7554/eLife.74255 35357307 PMC8970588

[bib58] Pincus, D. 2020. Regulation of Hsf1 and the heat shock response. Adv. Exp. Med. Biol. 1243:41–50. 10.1007/978-3-030-40204-4_3 32297210

[bib59] Powis, K., B. Schrul, H. Tienson, I. Gostimskaya, M. Breker, S. High, M. Schuldiner, U. Jakob, and B. Schwappach. 2013. Get3 is a holdase chaperone and moves to deposition sites for aggregated proteins when membrane targeting is blocked. J. Cell Sci. 126:473–483. 10.1242/jcs.112151 23203805 PMC3613179

[bib60] Prasad, R., C. Xu, and D.T.W. Ng. 2018. Hsp40/70/110 chaperones adapt nuclear protein quality control to serve cytosolic clients. J. Cell Biol. 217:2019–2032. 10.1083/jcb.201706091 29653997 PMC5987712

[bib61] Protter, D.S.W., and R. Parker. 2016. Principles and properties of stress granules. Trends Cell Biol. 26:668–679. 10.1016/j.tcb.2016.05.004 27289443 PMC4993645

[bib62] Riback, J.A., C.D. Katanski, J.L. Kear-Scott, E.V. Pilipenko, A.E. Rojek, T.R. Sosnick, and D.A. Drummond. 2017. Stress-triggered phase separation is an adaptive, evolutionarily tuned response. Cell. 168:1028–1040.e19. 10.1016/j.cell.2017.02.027 28283059 PMC5401687

[bib63] Rüdiger, S., J. Schneider-Mergener, and B. Bukau. 2001. Its substrate specificity characterizes the DnaJ co-chaperone as a scanning factor for the DnaK chaperone. EMBO J. 20:1042–1050. 10.1093/emboj/20.5.1042 11230128 PMC145471

[bib64] Sagot, I., and D. Laporte. 2019. Quiescence, an individual journey. Curr. Genet. 65:695–699. 10.1007/s00294-018-00928-w 30649583

[bib65] Samant, R.S., C.M. Livingston, E.M. Sontag, and J. Frydman. 2018. Distinct proteostasis circuits cooperate in nuclear and cytoplasmic protein quality control. Nature. 563:407–411. 10.1038/s41586-018-0678-x 30429547 PMC6707801

[bib66] Santagata, S., M.L. Mendillo, Y.C. Tang, A. Subramanian, C.C. Perley, S.P. Roche, B. Wong, R. Narayan, H. Kwon, M. Koeva, . 2013. Tight coordination of protein translation and HSF1 activation supports the anabolic malignant state. Science. 341:1238303. 10.1126/science.1238303 23869022 PMC3959726

[bib67] Santiago, A.M., D.L. Gonçalves, and K.A. Morano. 2020. Mechanisms of sensing and response to proteotoxic stress. Exp. Cell Res. 395:112240. 10.1016/j.yexcr.2020.112240 32827554 PMC7541750

[bib68] Schilke, B.A., and E.A. Craig. 2022. Essentiality of Sis1, a J-domain protein Hsp70 cochaperone, can be overcome by Tti1, a specialized PIKK chaperone. Mol. Biol. Cell. 33:br3. 10.1091/mbc.E21-10-0493 34935410 PMC9250385

[bib69] Seidel, M., N. Romanov, A. Obarska-Kosinska, A. Becker, N. Trevisan Doimo de Azevedo, J. Provaznik, S.R. Nagaraja, J.J.M. Landry, V. Benes, and M. Beck. 2023. Co-translational binding of importins to nascent proteins. Nat. Commun. 14:3418. 10.1038/s41467-023-39150-9 37296145 PMC10256725

[bib70] Shakya, V.P.S., W.A. Barbeau, T. Xiao, C.S. Knutson, M.H. Schuler, and A.L. Hughes. 2021. A nuclear-based quality control pathway for non-imported mitochondrial proteins. Elife. 10:e61230. 10.7554/eLife.61230 33734083 PMC7993989

[bib71] Shan, S.O. 2019. Guiding tail-anchored membrane proteins to the endoplasmic reticulum in a chaperone cascade. J. Biol. Chem. 294:16577–16586. 10.1074/jbc.REV119.006197 31575659 PMC6851334

[bib72] Shore, D., and B. Albert. 2022. Ribosome biogenesis and the cellular energy economy. Curr. Biol. 32:R611–R617. 10.1016/j.cub.2022.04.083 35728540

[bib73] Shore, D., S. Zencir, and B. Albert. 2021. Transcriptional control of ribosome biogenesis in yeast: Links to growth and stress signals. Biochem. Soc. Trans. 49:1589–1599. 10.1042/BST20201136 34240738 PMC8421047

[bib74] Sontag, E.M., F. Morales-Polanco, J.H. Chen, G. McDermott, P.T. Dolan, D. Gestaut, M.A. Le Gros, C. Larabell, and J. Frydman. 2023. Nuclear and cytoplasmic spatial protein quality control is coordinated by nuclear-vacuolar junctions and perinuclear ESCRT. Nat. Cell Biol. 25:699–713. 10.1038/s41556-023-01128-6 37081164 PMC12349969

[bib75] Sontag, E.M., R.S. Samant, and J. Frydman. 2017. Mechanisms and functions of spatial protein quality control. Annu. Rev. Biochem. 86:97–122. 10.1146/annurev-biochem-060815-014616 28489421

[bib76] Sun, S., and D. Gresham. 2021. Cellular quiescence in budding yeast. Yeast. 38:12–29. 10.1002/yea.3545 33350503 PMC8208048

[bib77] Sung, M.-K., J.M. Reitsma, M.J. Sweredoski, S. Hess, and R.J. Deshaies. 2016a. Ribosomal proteins produced in excess are degraded by the ubiquitin-proteasome system. Mol. Biol. Cell. 27:2642–2652. 10.1091/mbc.e16-05-0290 27385339 PMC5007085

[bib78] Sung, M.K., T.R. Porras-Yakushi, J.M. Reitsma, F.M. Huber, M.J. Sweredoski, A. Hoelz, S. Hess, and R.J. Deshaies. 2016b. A conserved quality-control pathway that mediates degradation of unassembled ribosomal proteins. Elife. 5:e19105. 10.7554/eLife.19105 PMC5026473 27552055

[bib79] Sutandy, F.X.R., I. Gößner, G. Tascher, and C. Münch. 2023. A cytosolic surveillance mechanism activates the mitochondrial UPR. Nature. 618:849–854. 10.1038/s41586-023-06142-0 37286597 PMC10284689

[bib80] Triandafillou, C.G., C.D. Katanski, A.R. Dinner, and D.A. Drummond. 2020. Transient intracellular acidification regulates the core transcriptional heat shock response. Elife. 9:e54880. 10.7554/eLife.54880 32762843 PMC7449696

[bib81] Tye, B.W., and L.S. Churchman. 2021. Hsf1 activation by proteotoxic stress requires concurrent protein synthesis. Mol. Biol. Cell. 32:1800–1806. 10.1091/mbc.E21-01-0014 34191586 PMC8684711

[bib82] Tye, B.W., N. Commins, L.V. Ryazanova, M. Wühr, M. Springer, D. Pincus, and L.S. Churchman. 2019. Proteotoxicity from aberrant ribosome biogenesis compromises cell fitness. Elife. 8:e43002. 10.7554/eLife.43002 30843788 PMC6453566

[bib83] Wallace, E.W., J.L. Kear-Scott, E.V. Pilipenko, M.H. Schwartz, P.R. Laskowski, A.E. Rojek, C.D. Katanski, J.A. Riback, M.F. Dion, A.M. Franks, . 2015. Reversible, specific, active aggregates of endogenous proteins assemble upon heat stress. Cell. 162:1286–1298. 10.1016/j.cell.2015.08.041 26359986 PMC4567705

[bib84] Wang, F., C. Chan, N.R. Weir, and V. Denic. 2014. The Get1/2 transmembrane complex is an endoplasmic-reticulum membrane protein insertase. Nature. 512:441–444. 10.1038/nature13471 25043001 PMC4342754

[bib85] Woolford, J.L. Jr., and S.J. Baserga. 2013. Ribosome biogenesis in the yeast Saccharomyces cerevisiae. Genetics. 195:643–681. 10.1534/genetics.113.153197 24190922 PMC3813855

[bib86] Work, J.J., and O. Brandman. 2021. Adaptability of the ubiquitin-proteasome system to proteolytic and folding stressors. J. Cell Biol. 220:e201912041. 10.1083/jcb.201912041 33382395 PMC7780722

[bib87] Xu, Y., D.E. Anderson, and Y. Ye. 2016. The HECT domain ubiquitin ligase HUWE1 targets unassembled soluble proteins for degradation. Cell Discov. 2:16040. 10.1038/celldisc.2016.40 27867533 PMC5102030

[bib88] Yagita, Y., E. Zavodszky, S.-Y. Peak-Chew, and R.S. Hegde. 2023. Mechanism of orphan subunit recognition during assembly quality control. Cell. 186:3443–3459.e24. 10.1016/j.cell.2023.06.016 37480851 PMC10501995

[bib89] Yanagitani, K., S. Juszkiewicz, and R.S. Hegde. 2017. UBE2O is a quality control factor for orphans of multiprotein complexes. Science. 357:472–475. 10.1126/science.aan0178 28774922 PMC5549844

[bib90] Yoo, H., C. Triandafillou, and D.A. Drummond. 2019. Cellular sensing by phase separation: Using the process, not just the products. J. Biol. Chem. 294:7151–7159. 10.1074/jbc.TM118.001191 30877200 PMC6509497

[bib91] Zavodszky, E., S.-Y. Peak-Chew, S. Juszkiewicz, A.J. Narvaez, and R.S. Hegde. 2021. Identification of a quality-control factor that monitors failures during proteasome assembly. Science. 373:998–1004. 10.1126/science.abc6500 34446601 PMC7611656

[bib92] Zheng, X., J. Krakowiak, N. Patel, A. Beyzavi, J. Ezike, A.S. Khalil, and D. Pincus. 2016. Dynamic control of Hsf1 during heat shock by a chaperone switch and phosphorylation. Elife. 5:e18638. 10.7554/eLife.18638 27831465 PMC5127643

[bib93] Zid, B.M., and E.K. O’Shea. 2014. Promoter sequences direct cytoplasmic localization and translation of mRNAs during starvation in yeast. Nature. 514:117–121. 10.1038/nature13578 25119046 PMC4184922

